# Breast Arterial Calcification is Associated with the Progression of Coronary Atherosclerosis in Asymptomatic Women: A Preliminary Retrospective Cohort Study

**DOI:** 10.1038/s41598-020-59606-y

**Published:** 2020-02-17

**Authors:** Yeonyee Elizabeth Yoon, Kyoung Min Kim, Wonjae Lee, Jong Soo Han, Eun Ju Chun, Soyeon Ahn, Sang Il Choi, Bo La Yun, Jung-Won Suh

**Affiliations:** 1 Departments of Cardiology, Seoul National University Bundang Hospital, Seongnam-si, Gyeonggi-do Republic of Korea; 2 Departments of Endocrinology, Seoul National University Bundang Hospital, Seongnam-si, Gyeonggi-do Republic of Korea; 3 Departments of Health Promotion Center, Seoul National University Bundang Hospital, Seongnam-si, Gyeonggi-do Republic of Korea; 4 Departments of Radiology, Seoul National University Bundang Hospital, Seongnam-si, Gyeonggi-do Republic of Korea; 5 Departments of Medical Research Collaborating Center, Seoul National University Bundang Hospital, Seongnam-si, Gyeonggi-do Republic of Korea; 6 Departments of Internal Medicine, Seoul National University College of Medicine, Seoul, Republic of Korea; 7 Departments of Family Medicine, Seoul National University College of Medicine, Seoul, Republic of Korea; 8 Departments of Radiology, Seoul National University College of Medicine, Seoul, Republic of Korea

**Keywords:** Cardiovascular diseases, Heart failure

## Abstract

We evaluated whether breast arterial calcification (BAC) is associated with the progression of coronary atherosclerosis in asymptomatic women. This retrospective observational cohort study analysed asymptomatic women from the BBC registry. In 126 consecutive women (age, 54.5 ± 7.0 years) who underwent BAC evaluation and repeated coronary computed tomography angiography (CCTA) examinations, the coronary arterial calcification score (CACS) and segment stenosis score (SSS) were evaluated to assess the progression of coronary arterial calcification (CAC) and coronary atherosclerotic plaque (CAP). CAC and CAP progression were observed in 42 (33.3%) and 26 (20.6%) women, respectively (median interscan time, 4.3 years), and were associated with the presence of BAC and a higher BAC score at baseline. Women with BAC demonstrated higher CAC and CAP progression rates and showed higher chances for CAC and CAP progression during follow-up (p < 0.001 for both). In multivariable analyses, the BAC score remained independently associated with both CAC and CAP progression rates after adjustment for clinical risk factors (β = 0.087, p = 0.029; and β = 0.020, p = 0.010, respectively) and with additional adjustment for baseline CACS (β = 0.080, p = 0.040; and β = 0.019, p = 0.012, respectively) or SSS (β = 0.079, p = 0.034; and β = 0.019, p = 0.011, respectively). Thus, BAC may be related to the progression of coronary atherosclerosis and its evaluation may facilitate decision-making.

## Introduction

Breast arterial calcification (BAC), which is commonly observed on screening mammography, represents medial calcification of the mammary arteries and is considered as a benign and incidental finding from an oncological perspective. However, at the same time, BAC has been reported as a women-specific risk marker for cardiovascular disease^1,2^. Evidence gathered over the past several decades has demonstrated associations between BAC presence and traditional cardiovascular risk factors, as well as an increased risk for adverse cardiac events in women with BAC^2–4^. Moreover, recent studies have reported that the presence of BAC is associated with coronary artery calcification (CAC) and coronary atherosclerotic plaque (CAP)^5,6^. Especially, the BBC study (Women Health Registry Study for Bone, Breast, and Coronary Artery Disease) demonstrated that BAC evaluation in asymptomatic women provides an independent and incremental value to clinical risk factors for the prediction of subclinical coronary atherosclerosis^6^.

If coronary atherosclerosis progression could be predicted in individual patients by evaluating BAC, in addition to clinical risk factors, clinical decision-making regarding further cardiac tests and preventive medication would be facilitated. Furthermore, given that millions of women undergo mammography, the simultaneous evaluation of breast cancer and cardiovascular disease risk has tremendous appeal. However, to date, whether BAC evaluation is associated with coronary atherosclerosis progression has not been studied. Therefore, in this retrospective cohort study, we aimed to evaluate whether the presence and severity of BAC on mammography is associated with coronary atherosclerosis progression in asymptomatic women.

## Materials and Methods

### Study design and participants

This retrospective study was approved by the institutional review board of Seoul National University Bundang Hospital of Seoul National University, and the requirement of informed consent was waived. All experiments were performed in accordance with the Declaration of Helsinki and relevant guidelines and regulations.

The study participants were recruited from the BBC study, which was a retrospective observational cohort study that assessed the predictive value of BAC and bone mineral density for the presence of subclinical coronary artery disease (CAD). A total of 2,100 consecutive asymptomatic women aged ≥40 years who underwent digital mammography, dual-energy X-ray absorptiometry, and coronary computed tomography angiography (CCTA) as part of a general health evaluation at the Health Promotion Center, Seoul National University Bundang Hospital, between 2011 and 2013 were included in the BBC study. As we previously described, although most Korean health check-up centers are currently trying to curb the use of CCTA in asymptomatic individuals^7^, Korean individuals have been able to undergo CCTA through a self-referral mechanism. The design, characteristics, and primary findings of the BBC study have previously been described^6^.

**Figure 1:**
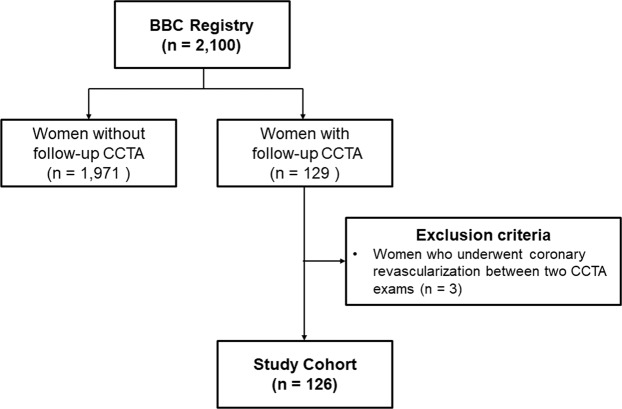
Flow diagram of the study population selection. BBC Registry, Women Health Registry for Bone, Breast, and Coronary Artery Disease; CCTA, coronary computed tomographic angiography.

From the retrospective observational cohort of the BBC study, we identified 129 women who underwent repeated CCTA examinations. After excluding 3 women who underwent coronary revascularisation between two CCTA examinations, 126 women were included in the final analysis (Fig. 1). Of these, 43 (34.1%) underwent symptom-driven clinically indicated CCTA examinations, while 83 (65.9%) underwent CCTA as a part of a general health evaluation.

### Digital mammography

As previously described, standard two-view (craniocaudal and mediolateral oblique) screening mammography was performed using a full-field digital mammography system (Brestige; Medi-future) and retrospectively evaluated by a breast radiologist, blinded to the CCTA results and clinical outcomes, using a 5-megapixel monitor and picture-archiving and communication system (Infinitt PACS®; Infinitt Healthcare)^6^. For women with BAC, the number, length, and density of BACs were evaluated as previously described^5,6^. The number of calcified vessels in both breasts was coded as 0 to 6 (if more than 6 calcified vessels were detected, 6 was recorded). The calcification involvement within the entire length of the calcified vessel with the longest calcification among all mammary arteries, was scored as 0 to 3 (0, none; 1, <1/3; 2, between 1/3 and 2/3; 3, >2/3). Similarly, the density of a calcified vessel in its densest segment was scored as 0 to 3 (0, none; 1, vessel wall calcification with clear visualisation of the lumen; 2, vessel wall calcification with clouding of the lumen; 3, vessel wall calcification without visualisation of the lumen). The BAC score (BACS) was calculated by summing these 3 numbers^5,6^.

### CCTA acquisition and analysis

As previously described, CCTA was performed using a 64-detector row computed tomography (CT) scanner (Brilliance 64; Philips Medical Systems, Best) and the images were transferred to an offline 3-dimensional workstation. CCTA images were independently analysed for CAC and CAP^6^. The CAC score (CACS) was measured using the Agatston scoring system^8^. CAP was defined as the presence of any clearly discernible atherosclerotic plaque lesion >1 mm^2^ that could be discriminated from the coronary artery in ≥2 independent image planes^9^. The segment stenosis score (SSS) was used as a measure of the overall CAP burden^10^. According to the modified American Heart Association 15-segment criteria^11^, each segment was graded from 0 to 2: 0, no CAP; 1, CAP with maximal diameter stenosis <50%; and 2, CAP with maximal diameter stenosis ≥50%. The scores of all 15 segments were summed to yield a SSS ranging from 0–30. CAC and CAP progression were defined as increase in the CACS and SSS, respectively, on follow-up CCTA compared to that on baseline CCTA. The CAC progression rate was calculated as the annualised difference between the square root of the baseline CACS and the square root of the follow-up CACS to minimise the effect of interscan variability^12,13^. The CAP progression rate was calculated as the annualised difference between the baseline and follow-up SSS.

### Clinical follow-up

Medical records were reviewed to assess the occurrence of cardiac death, nonfatal myocardial infarction, unstable angina, and revascularisation.

### Statistical analysis

All statistical analyses were performed using R statistical software (version 3.5.0, http://www.R-project.org/). Continuous variables are expressed as means and standard deviations, and categorical variables are expressed as proportions. Quantitative data were compared using the Student’s t, Kruskal-Wallis, χ^2^, and Fisher’s exact tests, as appropriate. The Kaplan-Meier method was used to visualise CAC and CAP progression, and the differences among groups with or without CAC or CAP progression were evaluated by the log-rank test. The date of the CCTA scan in which CAC or CAP progression was observed was assigned as the date of the event occurrence. Univariable and multivariable linear regression analyses were conducted to determine the effects of various characteristics on the annualised CAC and CAP progression. The results are expressed as the beta coefficient (β) and corresponding 95% confidence interval (CI). For the multivariable linear regression analysis, collinearity among all potential confounders was tested using variance inflation factors; values less than 2.0 were considered to indicate no collinearity. The independent association between the BACS and CAC or CAP progression rate was examined in several models: model 1, clinical risk factors + BACS; model 2, clinical risk factors + CACS + BACS; and model 3, clinical risk factors + SSS + BACS. We did not include CACS and SSS in the same model because of multicollinearity. The *t* value was used to estimate the contribution of each variable to the progression rates of CAC and CAP. A two-sided *p-*value < 0.05 was considered to represent a statistically significant difference.

### Statistics and biometry

One of the authors has significant statistical expertise.

### Informed consent

Written informed consent was waived by the Institutional Review Board.

### Ethical approval

Institutional Review Board approval was obtained.

### Study subjects or cohorts overlap

The study participants were recruited from the BBC study, which was a retrospective observational cohort study that assessed the predictive value of breast arterial calcification and bone mineral density for the presence of subclinical coronary artery disease.

## Results

### Baseline characteristics

Compared to those without follow-up CCTA (n = 1,971; mean age 52.3 ± 7.2 years), the women in the present study cohort (n = 126; mean age, 54.5 ± 7.0 years) were older and more likely to have hypertension and higher body mass index (Supplementary Table 1). They also were more likely to have CAC and CAP at baseline. However, BAC presence and score did not significantly differ between women with and without repeated CCTA examinations. Table 1 provides the baseline characteristics of the women in the present study cohort. At baseline, CAC and CAP were present in 27 (21.4%) and 39 (31.0%) women, respectively.

**Table 1:** Differences in baseline characteristics according to the progression of CAC and CAP.

	Entire Study Cohort	CAC progression		p-value	CAP progression		p-value
		(−)	(+)		(−)	(+)	
	(N = 126)	(N = 84)	(N = 42)		(N = 100)	(N = 26)	
Age, years	54.5 ± 7.0	53.2 ± 6.7	57.2 ± 6.9	0.002	53.6 ± 6.6	58.0 ± 7.8	0.004
Post-menopausal women, n (%)	98 (77.8%)	61 (72.6%)	37 (88.1%)	0.081	77 (77.0%)	21 (80.8%)	0.883
Parous woman	112 (88.9%)	73 (86.9%)	39 (92.9%)	0.438	90 (90.0%)	22 (84.6%)	0.669
Number of parity	2.1 ± 1.2	1.9 ± 1.0	2.5 ± 1.5	0.050	1.9 ± 1.0	2.5 ± 1.8	0.149
Hypertension, n (%)	30 (23.8%)	15 (17.9%)	15 (35.7%)	0.046	22 (22.0%)	8 (30.8%)	0.499
Diabetes mellitus, n (%)	5 (4.0%)	2 (2.4%)	3 (7.1%)	0.420	3 (3.0%)	2 (7.7%)	0.597
Hyperlipidaemia, n (%)	71 (56.3%)	42 (50.0%)	29 (69.0%)	0.066	53 (53.0%)	18 (69.2%)	0.206
Current smoking, n (%)	2 (1.8%)	1 (1.4%)	1 (2.6%)	1.000	1 (1.1%)	1 (4.2%)	0.896
Family history of CAD, n (%)	23 (29.9%)	16 (30.8%)	7 (28.0%)	1.000	20 (31.7%)	3 (21.4%)	0.660
Body mass index, kg/m^2^	23.3 ± 2.9	23.1 ± 2.9	23.8 ± 2.9	0.186	23.3 ± 3.0	23.6 ± 2.8	0.649
Systolic blood pressure, mmHg	112.7 ± 18.1	110.3 ± 17.7	117.4 ± 18.1	0.045	111.0 ± 17.6	119.0 ± 18.9	0.059
Diastolic blood pressure, mmHg	65.1 ± 11.8	63.0 ± 10.8	69.1 ± 12.8	0.006	63.6 ± 10.3	70.5 ± 15.5	0.040
Haemoglobin, g/dL	13.3 ± 1.2	13.2 ± 1.3	13.5 ± 0.9	0.229	13.3 ± 1.2	13.3 ± 0.8	0.952
Serum creatinine, mg/dL	0.7 ± 0.1	0.7 ± 0.1	0.7 ± 0.1	0.940	0.7 ± 0.1	0.7 ± 0.1	0.372
Fasting blood glucose, mg/dL	91.6 ± 23.8	87.7 ± 9.9	99.4 ± 37.9	0.058	89.6 ± 14.8	99.2 ± 43.4	0.464
HbA1c, %	5.7 ± 0.8	5.6 ± 0.4	6.1 ± 1.2	<0.001	5.6 ± 0.6	6.2 ± 1.3	0.001
Total cholesterol, mg/dL	207.3 ± 38.6	202.3 ± 34.9	217.2 ± 43.9	0.041	206.1 ± 39.3	211.9 ± 36.0	0.499
Triglyceride, mg/dL	99.5 ± 68.1	87.7 ± 51.0	123.1 ± 89.6	0.003	97.9 ± 71.4	105.7 ± 54.6	0.150
High-density lipoprotein, mg/dL	58.4 ± 13.3	59.2 ± 14.7	56.9 ± 10.1	0.462	58.2 ± 14.4	59.2 ± 8.2	0.354
Low-density lipoprotein, mg/dL	128.7 ± 33.7	124.0 ± 30.8	138.0 ± 37.5	0.028	127.7 ± 34.4	132.7 ± 31.1	0.411
Statin therapy after CCTA	13 (10.3%)	6 (7.1%)	7 (16.7%)	0.092	10 (10.0%)	3 (11.5%)	0.529
CAC presence, n (%)	27 (21.4%)	5 (6.0%)	22 (52.4%)	<0.001	16 (16.0%)	11 (42.3%)	0.008
CAC score	9.9 ± 40.4	1.3 ± 7.1	26.9 ± 66.6	<0.001	7.7 ± 40.7	18.2 ± 39.1	0.004
CAP presence, n (%)	39 (31.0%)	11 (13.1%)	28 (66.7%)	<0.001	27 (27.0%)	12 (46.2%)	0.100
SSS	0.6 ± 1.1	0.2 ± 0.7	1.3 ± 1.5	<0.001	0.4 ± 0.9	1.0 ± 1.7	0.044
BAC presence, n (%)	18 (14.3%)	6 (7.1%)	12 (28.6%)	0.003	9 (9.0%)	9 (34.6%)	0.003
BACS	0.8 ± 2.2	0.4 ± 1.5	1.6 ± 2.9	0.001	0.4 ± 1.5	2.2 ± 3.5	<0.001

Values are mean ± standard deviation or n (%).

BAC, breast arterial calcification; BACS, breast arterial calcification score; CAC, coronary arterial calcification; CACS, coronary artery calcification score; CAD, coronary artery disease; CAP, coronary atherosclerotic plaque; CCTA, coronary computed tomography angiography; SSS, segmental stenosis score.

### CAC and CAP progression

With a median interscan duration of 4.3 years (interquartile range, 3.2–5.0 years), CAC and CAP progression were observed in 42 (33.3%) and 26 (20.6%) women, respectively. Women who experienced CAC progression were significantly older and showed higher blood pressure, haemoglobin A1c (HbA1c), total cholesterol, triglyceride, and low-density lipoprotein (LDL) cholesterol than did women without CAC progression. They were also more likely to have CAC, CAP, and BAC at baseline (Table 1). Although women with CAP progression were older and had higher HbA1c than were those without CAP progression, the lipid profiles were not significantly different. They were also more likely to have CAC, CAP, and BAC at baseline.

### CAC and CAP changes based on BAC presence

**Figure 2:**
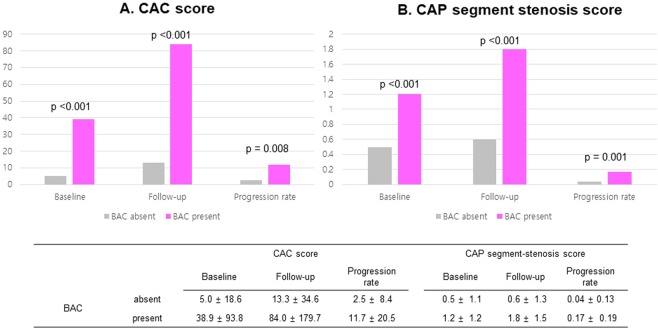
Progression of CAC (**A**) and CAP (**B**) according to the presence and absence of BAC. BAC, breast arterial calcification; CAC, coronary arterial calcification; CAP, coronary atherosclerotic plaque.

**Figure 3:**
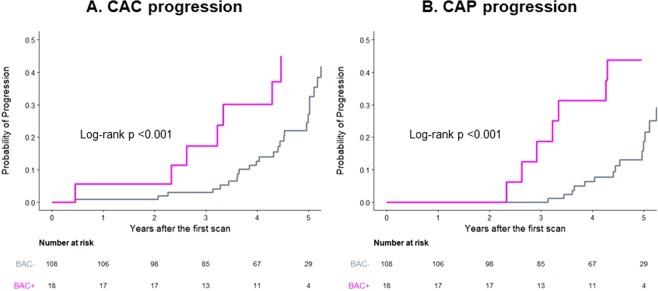
The cumulative proportion of CAC (**A**) and CAP (**B**) progression according to the presence and absence of BAC. BAC, breast arterial calcification; CAC, coronary arterial calcification; CAP, coronary atherosclerotic plaque.

**Figure 4:**
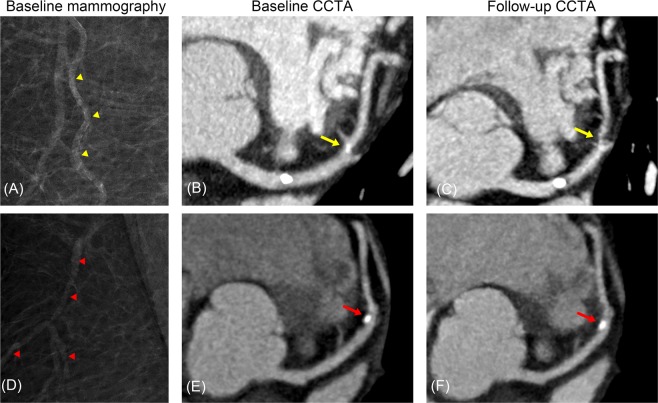
Representative cases. A 63-year-old asymptomatic woman with a BAC score of 5 (yellow arrow heads) on screening mammography (**A**) demonstrated calcified plaque without significant stenosis (percent diameter stenosis, 10–20%) at the proximal LAD and mixed plaque (percent diameter stenosis, 10–20%) at the mid LAD (yellow arrow). (**B**) Three years later, she was referred to the emergency department with chest pain and underwent CCTA, which demonstrated progression of the mid LAD lesion (percent diameter stenosis, 90%; yellow arrow). (**C**) Invasive angiography also demonstrated the tight stenosis of the mid LAD (percent diameter stenosis, 90%) and percutaneous coronary intervention was performed. A 51-year-old asymptomatic woman without evidence of BAC (red arrow heads) on screening mammography (**D**) demonstrated calcified plaque (percent diameter stenosis, 30%) at the mid LAD (red arrow). (**E**) Five years later, she visited the outpatient clinic due to epigastric pain and underwent CCTA, which demonstrated no change in the mid LAD lesion (red arrow). (**F**) BAC, breast arterial calcification; CCTA, coronary computed tomographic angiography; LAD, left anterior descending artery.

Women with BAC were more likely to have CAC at both baseline (55.6% vs. 15.7%; p < 0.001) and follow-up (77.8% vs. 29.6%; p < 0.001) than were women without BAC. Similarly, CAP was more frequently observed in women with BAC at both baseline (72.2% vs. 24.1%; p < 0.001) and follow-up (83.3% vs. 33.3%; p < 0.001). The CACS, SSS, and CAC and CAP progression rates stratified by BAC presence are shown in Fig. 2. CACS and SSS were significantly higher in women with BAC than in women without BAC at both baseline and follow-up. The progression rates of CAC and CAP were also higher in women with BAC than in women without BAC. The cumulative proportions of CAC and CAP progression stratified by BAC presence are shown in Fig. 3. Women with BAC at baseline had a significantly higher chance of CAC and CAP progression compared to that in women without BAC. Representative cases are shown in Fig. 4.

### Factors associated with CAC and CAP progression rates

Table 2 shows the results of the univariable linear regression analyses for CAC and CAP progression rates. The results of the multivariable linear regression analysis for the CAC progression rate are shown in Table 3. Among the clinical risk factors, age, number of parity, hypertension, current smoking, systolic blood pressure, serum creatinine, HbA1c, triglyceride, high-density lipoprotein cholesterol, LDL cholesterol, statin use at day 60 after the index CCTA were included in the multivariable analysis, after excluding variables showing multicollinearity. In models 1 and 2, HbA1c and BACS were independently associated with the CAC progression rate. In model 3, HbA1c, SSS, and BACS maintained independent associations with the CAC progression rate. The results of a similar multivariable linear regression analysis for the CAP progression rate are shown in Table 4. In models 1, 2, and 3, only the BACS maintained an independent association with the CAP progression rate.

**Table 2:** Univariable Linear Regression Analyses for CAC and CAP Progression Rates.

	CAC progression rate			CAP progression rate		
	β	95% CI	p	Β	95% CI	p
Age, years	0.022	0.002–0.041	0.034	0.007	0.003–0.010	<0.001
Post-menopausal women	0.169	−0.169–0.507	0.323	0.029	−0.034–0.092	0.364
Parous woman	0.254	−0.193–0.700	0.263	−0.034	−0.117–0.050	0.429
Number of parity	0.129	0.015–0.243	0.027	0.018	−0.003–0.040	0.095
Hypertension	0.296	−0.031–0.622	0.076	0.014	−0.048–0.075	0.666
Diabetes mellitus	1.448	0.773–2.122	<0.001	0.064	−0.071–0.198	0.350
Hyperlipidaemia	0.237	−0.044–0.518	0.097	0.051	−0.002–0.103	0.058
Current smoking	0.244	−0.939–1.427	0.684	0.039	−0.170–0.249	0.709
Family history of CAD	−0.194	−0.600–0.213	0.346	−0.035	−0.111–0.040	0.350
Body mass index, kg/m^2^	0.044	−0.004–0.091	0.072	0.005	−0.004–0.014	0.269
Systolic blood pressure, mmHg	0.010	0.003–0.018	0.009	0.002	0.000–0.003	0.023
Diastolic blood pressure, mmHg	0.011	−0.001–0.023	0.063	0.003	0.001–0.006	0.002
Haemoglobin, g/dL	0.109	−0.010–0.229	0.072	0.004	−0.018–0.027	0.719
Serum creatinine, mg/dL	0.085	−1.607–1.777	0.921	0.128	−0.188–0.443	0.425
Fasting blood glucose, mg/dL	0.014	0.008–0.019	<0.001	0.001	−0.001–0.002	0.319
HbA1c, %	0.497	0.343–0.651	<0.001	0.040	0.008–0.072	0.016
Total cholesterol, mg/dL	0.002	−0.002–0.005	0.415	0.000	−0.000–0.001	0.365
Triglyceride, mg/dL	0.002	0.000–0.004	0.020	0.000	−0.000–0.000	0.316
High-density lipoprotein, mg/dL	−0.009	−0.019–0.002	0.111	0.000	−0.002–0.002	0.929
Low-density lipoprotein, mg/dL	0.002	−0.002–0.006	0.282	0.000	−0.000–0.001	0.333
Statin therapy after CCTA	0.177	−0.285–0.640	0.449	0.002	−0.085–0.089	0.963
CAC presence	0.607	0.281–0.933	0.000	0.128	0.068–0.188	<0.001
CACS	0.005	0.001–0.008	0.008	0.001	0.000–0.001	0.075
CAP presence	0.642	0.359–0.925	<0.001	0.068	0.012–0.124	0.017
SSS	0.335	0.224–0.446	<0.001	0.032	0.009–0.055	0.006
BAC presence	0.482	0.089–0.876	0.017	0.129	0.057–0.200	0.001
BACS	0.074	0.010–0.138	0.024	0.023	0.116–0.035	<0.001

CI, confidence interval; other abbreviations are the same as those in Table 1.

**Table 3:** Multivariable Linear Regression Analysis for the CAC Progression Rate.

	Model 1 (Clinical RFs + BACS)				Model 2 (Clinical RFs + CACS + BACS)				Model 3 (Clinical RFs + Segment stenosis score + BACS)			
	β	95% CI	t	P	β	95% CI	t	p	β	95% CI	t	p
Age, years	−0.015	−0.040–0.011	−1.158	0.250	−0.019	−0.045–0.006	−1.497	0.137	−0.023	−0.047–0.002	−1.858	0.066
Number of parity	0.060	−0.077–0.198	0.871	0.386	0.044	−0.093–0.181	0.641	0.523	0.052	−0.077–0.181	0.806	0.422
Hypertension, n (%)	0.120	−0.237–0.478	0.669	0.505	0.107	−0.246–0.460	0.602	0.549	0.155	−0.181–0.490	0.914	0.363
Current smoking, n (%)	0.196	−0.846–1.237	0.373	0.710	0.174	−0.855–1.203	0.336	0.738	0.259	−0.718–1.236	0.527	0.600
Systolic blood pressure, mmHg	0.006	−0.002–0.014	1.421	0.158	0.006	−0.002–0.014	1.530	0.129	0.003	−0.005–0.011	0.783	0.435
Serum creatinine, mg/dL	0.398	−1.235–2.032	0.484	0.630	0.575	−1.050–2.199	0.702	0.485	−0.049	−1.597–1.500	−0.062	0.950
HbA1c, %	0.493	0.323–0.663	5.736	<0.001	0.497	0.329–0.666	5.855	<0.001	0.431	0.268–0.594	5.241	<0.001
High-density lipoprotein, mg/dL	−0.007	−0.018–0.003	−1.365	0.175	−0.006	−0.017–0.004	−1.192	0.236	−0.006	−0.016–0.004	−1.222	0.224
Low-density lipoprotein, mg/dL	0.002	−0.003–0.006	0.738	0.462	0.001	−0.003–0.005	0.581	0.563	0.001	−0.003–0.005	0.559	0.577
Statin therapy after CCTA	−0.021	−0.445–0.402	−0.100	0.921	−0.051	−0.471–0.368	−0.243	0.809	−0.170	−0.574–0.234	−0.834	0.406
CACS					0.003	0.000–0.007	1.875	0.064				
SSS									0.238	0.116–0.361	3.864	<0.001
BACS	0.087	0.009–0.164	2.221	0.029	0.080	0.004–0.157	2.076	0.040	0.079	0.006–0.151	2.146	0.034

Abbreviations are the same as those in Tables 1 and 2.

**Table 4:** Multivariable Linear Regression Analysis for the CAP Progression Rate.

	Model 1 (Clinical RFs + BACS)				Model 2 (Clinical RFs + CACS + BACS)				Model 3 (Clinical RFs + Segment stenosis score + BACS)			
	β	95% CI	t	P	β	95% CI	t	p	β	95% CI	t	p
Age, years	0.002	−0.003–0.007	0.916	0.362	0.002	−0.003–0.007	0.779	0.438	0.002	−0.003–0.007	0.775	0.440
Number of parity	0.004	−0.023–0.030	0.291	0.772	0.003	−0.024–0.030	0.207	0.836	0.004	−0.023–0.030	0.266	0.791
Hypertension, n (%)	−0.026	−0.095–0.043	−0.746	0.457	−0.027	−0.096–0.042	−0.769	0.444	−0.025	−0.094–0.045	−0.704	0.483
Current smoking, n (%)	0.073	−0.128–0.274	0.721	0.472	0.072	−0.130–0.273	0.705	0.483	0.076	−0.126–0.277	0.745	0.458
Systolic blood pressure, mmHg	0.001	0.000–0.003	1.793	0.076	0.001	0.000–0.003	1.818	0.072	0.001	0.000–0.003	1.620	0.108
Serum creatinine, mg/dL	0.122	−0.193–0.437	0.767	0.445	0.134	−0.185–0.452	0.834	0.406	0.104	−0.216–0.423	0.644	0.521
HbA1c, %	0.031	−0.002––0.063	1.840	0.069	0.031	−0.002–0.064	1.851	0.067	0.028	−0.006–0.062	1.651	0.102
High-density lipoprotein, mg/dL	0.000	−0.002–0.002	0.126	0.900	0.000	−0.002–0.002	0.189	0.851	0.000	−0.002–0.002	0.172	0.864
Low-density lipoprotein, mg/dL	0.000	−0.001–0.001	0.378	0.706	0.000	−0.001–0.001	0.319	0.750	0.000	−0.001–0.001	0.332	0.741
Statin therapy after CCTA	−0.023	−0.105–0.059	−0.560	0.577	−0.025	−0.107–0.057	−0.606	0.546	−0.029	−0.113–0.054	−0.693	0.490
CACS					0.000	0.000–0.001	0.645	0.520				
SSS									0.010	−0.016–0.035	0.762	0.448
BACS	0.020	0.005–0.035	2.629	0.010	0.019	0.004–0.034	2.556	0.012	0.019	0.004–0.035	2.577	0.011

Abbreviations are the same as those in Tables 1 and 2.

### Clinical outcomes based on BAC presence

A total of 2 adverse cardiac events (1 nonfatal myocardial infarction and 1 unstable angina) were observed in patients with BAC, whereas no adverse cardiac events occurred among patients without BAC (thus, Cox regression analysis was unavailable). Both women who experienced adverse cardiac events also demonstrated CAC and CAP progression. The annualised rate of adverse cardiac events was 2.5% and 0% in women with and without BAC, respectively (log-rank p < 0.001).

## Discussion

We previously reported that BAC evaluation in asymptomatic women provides an independent and incremental value over conventional clinical risk factors for the prediction of subclinical coronary atherosclerosis^6^. Here, although the study population was limited to those with repeated CCTA examinations, we have additionally demonstrated that BAC presence and score are significantly associated with the progression of subclinical coronary atherosclerosis, as evidenced by increased CACS and SSS. In addition, the BACS is independently associated with the annualised progression of CAC and CAP.

BAC, incidentally observed at screening mammography, has been considered to be an insignificant finding without an increased risk of breast cancer. However, evidence that BAC presence is associated with an increased risk of cardiovascular disease morbidity and mortality is currently accumulating^2–4^. Nevertheless, limited data is currently available regarding the relationship between BAC and CAD, and the results are inconsistent^2^. With the exception of only one prospective study^14^, these previous studies retrospectively analysed symptomatic women who underwent invasive coronary angiography; thus, the study populations were necessarily limited to a small number of patients with suspected CAD^2^. Recently, Margolies *et al*. studied 292 women with digital mammography who also underwent non-gated chest CT^5^. Although CAC was assessed in a semiquantitative manner, the investigators showed a strong association between BAC and CAC. However, they failed to demonstrate an incremental predictive value of BAC over the conventional risk stratification algorithm, potentially due to the small number of study participants. In contrast, the BBC study, which included a large number of asymptomatic women, reported an association between BAC and subclinical CAD, including both CAC and CAP, as assessed on CCTA^6^. Additionally, BAC was shown to provide an independent and incremental predictive value over the conventional risk stratification algorithm. Therefore, BAC is currently expected to be useful for improving risk stratification, in a manner similar to that for CAC in previous studies^2,15^. Nevertheless, until recently, whether BAC presence and severity could also predict coronary atherosclerosis progression had never been evaluated. In the present preliminary study, we found an independent association between the BACS and the progression rates of CAC and CAP. Although the current analysis is based on a relatively small number of women with repeated CCTA examinations, which may raise concerns regarding generalizability, these results provide important clues regarding the association between BAC and the progression of coronary atherosclerosis.

Previous studies have demonstrated that CAC or CAP progression detected by serial CCTA is independently associated with adverse cardiovascular events^16–19^. Given that atherosclerosis is a dynamic process, CAC and CAP progression might provide insight into ongoing disease activity. However, although many experts have suggested that repeated testing provides a large amount of information regarding the progression of coronary atherosclerosis, no current guidelines advocate for more than one CAC scoring or CCTA for CAP evaluation. Additionally, the increasing cost of cardiovascular health care has become daunting. In contrast, current clinical practice guidelines recommend annual mammographic screening in asymptomatic women^20^, although the recommended age varies according to demographic characteristics and the available medical resources^21^. Thus, millions of women undergo mammography each year for the screening of breast cancer^22^. However, important information regarding the presence and severity of BAC is often omitted from the mammography report since it is considered as an insignificant finding without increased risk for breast cancer. Our study results suggest that BAC should be included in the mammography report and be treated as any other clinically relevant incidental finding. Of course, large randomised controlled trials are required before integrating BAC evaluation in risk stratification and personalised targeted preventive strategies.

Although evidence supporting the association of BAC with an increased risk for cardiovascular disease is accumulating, there remains uncertainty regarding the exact causal and pathophysiologic mechanism. While intimal calcification is commonly associated with atherosclerotic plaque, BAC is mostly (but not entirely) medial calcification^15^. Until the late twentieth century, medial calcification was considered rather benign because of the absence of stenotic lesions; however, it is currently recognised as a key negative predictor of cardiovascular morbidity and mortality^23^. It is not well understood why BAC predicts the presence and progression of subclinical coronary atherosclerosis. BAC may represent long-term exposure to shared cardiovascular risk factors, or it may be indicative of medial calcification co-existing in other vascular beds. Medial calcifications may lead to cardiovascular disease through increased arterial stiffness^24,25^. The decreased distensibility may contribute to higher peak pressure in distal vessels, leading to damage and remodelling, and an aggravation of ischaemia by co-existing atherosclerosis. Additionally, increased stiffness in large arteries may exacerbate atherosclerosis via altered blood flow characteristics^15^. As the BACS remained independently associated with CAC and CAP progression rates in the present study, further investigations are critical to deciphering the interactions between BAC and coronary atherosclerosis.

The major limitations of the present study are its observational retrospective design and the relatively small number of study participants, which subjected the study to many selection biases. Specifically, only 126 women of the 2,100 women in the BBC registry underwent repeated CCTA examinations. Although we were able to populate the BBC registry, because CCTA has been used as a screening tool through a self-referral mechanism in Korea, CCTA is not currently indicated in asymptomatic individuals^7^. Thus, it is not surprising that only a small number of women from the BBC registry underwent repeated CCTA examinations. Caution must be taken in applying the current preliminary results to the general population. Nevertheless, the current study provides valuable clues regarding the association between BAC and the progression of CAC and CAP that set the stage for an outcome trial, which is required to evaluate whether BAC evaluation on screening mammography translates into long-term clinical benefits. Additionally, in this retrospective study, CCTA follow-up was not guided by a specific study protocol. Therefore, the interscan duration varied among study participants; in an observational study, such effects are inevitable. To minimise the effect of variations in the interscan duration, we evaluated the association between BAC and annualised CAC and CAP progression (CAC and CAP progression rates). Finally, the CAP burden was estimated using the SSS instead of a volumetric measure of plaque because not all CCTA imaging data were stored at a sufficient level for plaque volumetry. Therefore, prospective studies are needed to determine whether BAC is predictive of increased CAP volume, and if so, whether it is also associated with changes in plaque composition.

## Conclusions

BAC, which is currently suggested as a potential woman-specific risk marker for CAD, is also related to the progression of coronary atherosclerosis as evidenced by CCTA. Especially, the BACS is independently associated with the annualised progression of CAC and CAP. Although these findings support the value of BAC in identifying asymptomatic women at an increased risk for future cardiovascular disease without additional cost and radiation exposure, caution must be exercised in applying the current preliminary results to the general population considering the relatively small number of study participants. Further studies are warranted to determine whether the evaluation of BAC in asymptomatic women predicts the progression of coronary atherosclerosis and translates into long-term clinical benefits.

## Supplementary information

Supplementary table.
