# Supplementary material for: Breast Arterial Calcification is Associated with the Progression of Coronary Atherosclerosis in Asymptomatic Women: A Preliminary Retrospective Cohort Study
Source: Sci Rep. 2020 Feb 17;10:2755. doi: 10.1038/s41598-020-59606-y (PMC7026413; doi:10.1038/s41598-020-59606-y)
Supplement: Supplementary file 1 — Supplementary table. [file 41598_2020_59606_MOESM1_ESM.docx]

**Breast Arterial Calcification is Associated with the Progression of Coronary Atherosclerosis in Asymptomatic Women: A Preliminary Retrospective Cohort Study**

Yeonyee Elizabeth Yoon^1, 6^, Kyoung Min Kim^2, 6^, Wonjae Lee^1, 6^, Jong Soo Han^3, 7^, Eun Ju Chun^4, 8^, Soyeon Ahn^5^, Sang Il Choi^4, 8^ ^*^Bo La Yun^4, 8^, ^*^Jung-Won Suh^1, 6^

Departments of ^1^Cardiology, ^2^Endocrinology, ^3^Health Promotion Center, ^4^Radiology, and ^5^Medical Research Collaborating Center, Seoul National University Bundang Hospital, Seongnam-si, Gyeonggi-do, Republic of Korea;

Departments of ^6^Internal Medicine, ^7^Family Medicine, and ^8^Radiology, Seoul National University College of Medicine

**^*^Corresponding author**

**Address for correspondence:**

Bo La Yun, MD, PhD

Department of Radiology, Seoul National University Bundang Hospital, 82 Gumi-ro 173 Beon-gil, Bundang-gu, Seongnam, Korea

Tel: +82-31-787-7633, Fax: +82-31-787-4011, E-mail: yunbola@gmail.com

and

Jung-Won Suh, MD, PhD

Department of Cardiology, Seoul National University Bundang Hospital

82 Gumi-ro 173 Beon-gil, Bundang-gu, Seongnam, Korea

Tel: +82-31-787-7016, Fax: +82-31-787-4051, E-mail: [dasome2@snu.ac.kr](mailto:dasome2@snu.ac.kr)

**Supplementary Table 1. Demographics and comparison of women with and without follow-up CCTA**

|  | **Women without follow-up CCTA** | **Study Cohort** | p |
| --- | --- | --- | --- |
|  | **(N=1971)** | **(N=126)** |  |
| Age, years | 52.3 ± 7.2 | 54.5 ± 7.0 | 0.001 |
| Post-menopausal, n (%) | 1220 (61.9%) | 98 (77.8%) | <0.001 |
| Parous, n (%) | 1695 (86.0%) | 112 (88.9%) | 0.436 |
| Number of parity | 1.9 ± 1.0 | 2.1 ± 1.2 | 0.191 |
| Hypertension, n (%) | 288 (14.6%) | 30 (23.8%) | 0.008 |
| Diabetes mellitus, n (%) | 81 (4.1%) | 5 (4.0%) | 1.000 |
| Hyperlipidemia, n (%) | 1082 (54.9%) | 71 (56.3%) | 0.822 |
| Current smoking, n (%) | 68 (4.0%) | 2 (1.8%) | 0.345 |
| Family history of CAD, n (%) | 224 (20.0%) | 23 (29.9%) | 0.055 |
| Body mass index, kg/m^2^ | 22.7 ± 3.0 | 23.3 ± 2.9 | 0.003 |
| Systolic blood pressure, mmHg | 110.5 ± 15.7 | 112.7 ± 18.1 | 0.299 |
| Diastolic blood pressure, mmHg | 63.6 ± 10.2 | 65.1 ± 11.8 | 0.232 |
| Hemoglobin, g/dL | 13.3 ± 1.2 | 13.3 ± 1.2 | 0.432 |
| Serum creatinine, mg/dL | 0.7 ± 0.1 | 0.7 ± 0.1 | 0.570 |
| Fasting blood glucose, mg/dL | 88.8 ± 15.0 | 91.6 ± 23.8 | 0.137 |
| HbA1c, % | 5.6 ± 0.6 | 5.7 ± 0.8 | 0.064 |
| Total cholesterol, mg/dL | 202.0 ± 35.1 | 207.3 ± 38.6 | 0.179 |
| Triglyceride, mg/dL | 91.3 ± 56.9 | 99.5 ± 68.1 | 0.183 |
| HDL cholesterol, mg/dL | 59.6 ± 13.9 | 58.4 ± 13.3 | 0.373 |
| LDL cholesterol, mg/dL | 123.6 ± 32.2 | 128.7 ± 33.7 | 0.105 |
| CAC presence, n (%) | 206 (10.5%) | 27 (21.4%) | <0.001 |
| CAC score | 10.0 ± 97.6 | 9.9 ± 40.4 | <0.001 |
| CAP presence, n (%) | 286 (14.5%) | 39 (31.0%) | <0.001 |
| Segment-stenosis score | 0.3 ± 1.0 | 0.6 ± 1.1 | <0.001 |
| BAC presence, n (%) | 181 (9.2%) | 18 (14.3%) | 0.082 |
| BAC score | 0.5 ± 1.7 | 0.8 ± 2.2 | 0.061 |

Values are mean ± standard deviation or n (%).

BAC, breast arterial calcification; CAC, coronary arterial calcification; CAD, coronary artery disease; CAP, coronary atherosclerotic plaque; CCTA, coronary computed tomography angiography; HbA1c, hemoglobin A1c; HDL, high-density lipoprotein; LDL, low-density lipoprotein.
